# Pharmacogenetics to Avoid Adverse Reactions in Cardiology: Ready for Implementation?

**DOI:** 10.3390/jpm11111180

**Published:** 2021-11-11

**Authors:** Xandra García-González, Sara Salvador-Martín

**Affiliations:** Instituto de Investigación Sanitaria Gregorio Marañón (IiSGM), Hospital General Universitario Gregorio Marañón, 28007 Madrid, Spain; sara.salvador@iisgm.com

**Keywords:** pharmacogenetics, cardiology, adverse events, guidelines

## Abstract

Cardiovascular Diseases (CVs) are one of the main causes of mortality and disability around the world. Advances in drug treatment have greatly improved survival and quality of life in the past decades, but associated adverse events remain a relevant problem. Pharmacogenetics can help individualize cardiovascular treatment, reducing associated toxicities and improving outcomes. Several scientific societies and working groups periodically review available studies and provide consensus recommendations for those gene-drug pairs with a sufficient level of evidence. However, these recommendations are rarely mandatory, and the indications on how to adjust treatment can vary between different guidelines, which limits their clinical applicability. The aim of this review is to compile, compare and discuss available guidelines and recommendations by the main Pharmacogenetics Consortiums (Clinical Pharmacogenetics Implementation Consortium (CPIC); Dutch Pharmacogenetics Working Group (DPWG); the French Network of Pharmacogenetics (Réseau national de pharmacogénétique (RNPGx) and The Canadian Pharmacogenomics Network for Drug Safety (CPNDS) regarding how to apply pharmacogenetic results to optimize pharmacotherapy in cardiology. Pharmacogenetic recommendations included in European or American drug labels, as well as those included in the European Society of Cardiology (ESC) and the American College of Cardiology (ACC) and the American Heart Association (AHA) treatment guidelines are also discussed.

## 1. Introduction

Cardiovascular Diseases (CVDs) are one of the main public health challenges of our time. They are currently the leading cause of death in the world and a major contributor to disability [1]. They include numerous entities among which Ischemic Heart Disease (IHD) and stroke are the most common.

CVDs were responsible for 18.6 million deaths in 2019 and prevalent cases reached 523 million. The global trends for Disability-Adjusted Life Years (DALYs) and years of life lost also increased significantly, and years lived with disability have almost doubled in the last three decades [2].

Thankfully, patient life expectancy and quality of care has significantly improved over the years thanks to prevention strategies, advances in surgery and other intervention techniques and of course, drug treatment. Anticoagulant and antiplatelet agents, beta-blockers, antihypertensive drugs, and lipid-lowering therapies are commonly used for the treatment and prevention of CVDs, accompanied by the modification of behavioral risk factors, such as tobacco and alcohol use, diet, and physical activity.

Despite their proven effectiveness and safety, drugs used in cardiology, as any drug, can also cause significant adverse reactions. Different factors influence interindividual variability in drug response and tolerance, but one of the most relevant is undoubtedly genetic variability [3].

The association between genetic variations and drug effectiveness and safety have been studied, although only a few are now being translated to clinical practice [4]. Several Scientific Societies and working groups periodically review available studies and provide consensus recommendations for those drug-gene pairs with a sufficient level of evidence to consider therapy modifications according to patient genotype. The Clinical Pharmacogenetics Implementation Consortium (CPIC) is probably the most influential one, but national guidance is also provided by the Dutch Pharmacogenetics Working Group (DPWG), the French Network of Pharmacogenetics (RNPGx) within the French Society of Pharmacology and Therapeutics (SFPT) and the Canadian Pharmacogenomics Network for Drug Safety (CPNDS) amongst others [5,6,7,8]. The Spanish Society of Pharmacogenetics and Pharmacogenomics has also included in its strategic plan the publication of practice guidelines for relevant drug-gene pairs [9]. Recently, the European Society of Cardiology issued a position statement on the role of Pharmacogenomics in contemporary cardiovascular therapy [10]. However, in most cases there are no official recommendations provided by the drug agencies. This together with the fact that recommendations from different Societies often differ from one another, makes the implementation into clinical practice challenging.

The aim of this review is to compile, compare and discuss available guidelines and recommendations regarding how to apply pharmacogenetic results to optimize pharmacotherapy in cardiology.

Reviewing the evidence supporting pharmacogenetic testing in cardiovascular medicine including available clinical trials that constitute the basis for the recommendations included in these guidelines falls beyond the scope of this article. An excellent review on this issue has been recently published by Duarte et al. [11].

## 2. Cardiovascular Drugs in Pharmacogenetics Guidelines

The most recent guidelines and publications by the Clinical Pharmacogenetics Implementation Consortium (CPIC); Dutch Pharmacogenetics Working Group (DPWG); the French Network of Pharmacogenetics (Réseau national de pharmacogénétique (RNPGx) and The Canadian Pharmacogenomics Network for Drug Safety (CPNDS) were reviewed and compared for drugs pertaining to therapeutic groups B01 ANTITHROMBOTIC AGENTS and C CARDIOVASCULAR SYSTEM of the Anatomical Therapeutic Chemical Classification System (ATC) of the World Health Organization (WHO) [12]. Table 1 includes all drug-gene pairs in these categories mentioned in the guidelines until 30 September 2021. The left row includes those drug-gene pairs for which a therapeutic recommendation (action recommended based on patient genotype such us dose adjustment or use of alternative treatment) has been issued by at least one of these organizations. Currently, it includes those drug-gene pairs that are reviewed but considered not to be a relevant drug-gene interaction at this time based on available evidence.

Additionally, the European Medicines Agency (EMA) and FDA drug labels were reviewed in search of any recommendations based on patient genotype. Any pharmacogenetic recommendations included in relevant treatment guidelines, such as those published by the European Society of Cardiology (ESC), the American College of Cardiology (ACC) and the American Heart Association (AHA) are also discussed below.

By 30 September 2021, 25 cardiovascular drug-gene pairs had been reviewed by at least one of the main groups issuing Pharmacogenetics guidelines (CPIC, DPWG, RNPGx and CPNDS). Eleven drug-gene pairs had therapeutic recommendations published by at least one of these groups. In more than half of the cases (6/11), therapeutic recommendations were made by just one of these groups, in one case (Acenocoumarol-VKORC1) recommendations were made by two groups and in two cases (Clopidogrel-CYP2C19, Simvastatin-SLCOB1 and Warfarin-VKORC1) by three of the four groups. Warfarin-CYP2C9 was the only drug-gene pair for which all four groups have issued therapeutic recommendations.

DPWG is by far the group that reviewed more cardiovascular drug-gene pairs [23] and also the one that has issued more therapeutic recommendations [10].

Published recommendations on how to avoid adverse reactions and increase treatment effectiveness based on patient genotype for drugs in the following therapeutic groups: anticoagulants, antiplatelet, statins, beta-blockers and antiarrhythmic, are reviewed below.

### 2.1. Anticoagulants

Three drugs in the ATC group B01AA Vitamin K antagonists are reviewed in the guidelines: warfarin, acenocoumarol and phenprocoumon. Different drug-gene pairs are reviewed and published recommendations vary between groups (Table 2): Warfarin-CYP2C9 is the drug-gene pair considered essential by the four groups; the CPIC, DPWG, RNPGx and CPNDS recommend therapeutic adjustment. Warfarin-VKORC1 has additional recommendations (always combined with CYP2C9 genotype) by CPIC, DPW and RNPGx. The CPIC is the only group that considers CYP4F2 genotype to further adjust warfarin dosage. Specific recommendations for other coumarin derivatives (acenocoumarol and phenprocoumon) and VKORC1 are only available by the DPWG, although these drugs are also mentioned in the RNPGx guidelines.

Coumarin derivatives, warfarin, acenocoumarol and phenprocoumon are Vitamin K antagonists (VKAs) used to prevent or treat thromboembolism. The dose of coumarin required to maintain an International Normalized Ratio (INR) between 2.0 and 3.0 is highly variable between individuals due to the narrow therapeutic window and significant inter- and intra-individual variability in a response that may be associated with over-anticoagulation (and thus risk of bleeding) or resistance to treatment.

#### Warfarin

Common genetic variants in CYP2C9, VKORC1, CYP4F2, in addition to known non-genetic factors, account for 50–60% of the variability in warfarin dosage [10].

- Warfarin and CYP2C9

CYP2C9 is the enzyme primarily responsible for the metabolic clearance of the S-warfarin [42].

In the Caucasian population, there are two main genetic variants associated with deficient CYP2C9 activity: polymorphism rs1799853 (CYP2C9*2 or c.430C>T) and rs1057910 (CYP2C9*3 or c.1075A>C) [43]. CYP2C9 allele frequencies differ between racial/ethnic groups; in fact, these alleles are significantly less prevalent in African-American and Asian populations [44].

The polymorphisms CYP2C9*2 and *3 compromise S-warfarin metabolism by 30–40% and 80–90%, respectively [44]. Compared to patients homozygous for CYP2C9*1, individuals with one or two copies of CYP2C9*2 or *3 may have an increased risk of bleeding when treated with warfarin as compared to patients with two normally- functioning alleles [45]. They require lower doses to achieve similar levels of anticoagulation and need more time to achieve a stable INR [44].

Other CYP2C9 alleles (CYP2C9*5, *6, *8 and *11), especially in the African population, are also associated with decreased CYP2C9 enzyme function and contribute to the variability of warfarin doses, requiring lower doses [46].

- Warfarin and VKORC1

VKORC1 encodes a key enzyme of the vitamin K cycle and is the pharmacological target of coumarinic anticoagulants, whose inhibitory action blocks vitamin K-dependent coagulation factors (factors II, VI, IX and X).

The c.−1639G>A variant (rs9923231) results in altered warfarin sensitivity, lower VKORC1 expression, and lower warfarin dose requirement during long-term treatment [47,48].

The −1639 AA genotype results in an increased sensitivity to warfarin. This results in an increased risk of an excessively severe inhibition of blood coagulation (INR > 4) during the first month of treatment, while the −1639 AG genotype results in a reduction in the required dose and an increase in the risk of an excessively severe inhibition of blood clotting during the first month of treatment. However, the effect is small and GA is the most common genotype, meaning that the standard treatment will primarily be based on patients with this genotype. As with the CYP2C9 variants, the frequency of the −1639G>A variant differs among ethnic groups, occurring most frequently in Asians.

- Warfarin and CYP4F2

CYP4F2 is an enzyme involved in vitamin K metabolism. It acts as an important counterpart of VKORC1 to limit excessive vitamin K accumulation [49]. CYP4F2 activity is decreased in the presence of the *3 allele (c.1297G>A; p.Val433Met; rs2108622). This *3 allele is associated with a higher bleeding risk and therefore, with lower warfarin dose requirements than the *1 allele to obtain the same anticoagulant response in European and Asian populations [50,51]. Moreover, inclusion of this CYP4F2 variant in warfarin dosing algorithms that included CYP2C9, VKORC1, and clinical factors improved the accuracy of dose prediction [50].

Both the DPWG and CPIC provide recommendations for CYP2C9 and VKORC1 gene pairs and warfarin. The CPIC calculates the recommended daily dosage for warfarin in mg/day based on specific algorithms including genotype (VKORC1−1639G>A, CYP2C9*2 and CYP2C9*3 alleles) and clinical variables that influence the response to warfarin [52,53]. Importantly, neither published algorithm includes the CYP2C9*5, *6, *8, *11, rs12777823 variant or the CYP4F2*3 allele, although, the CPIC provides separate guidance for patients of African and non-African descent. For populations of non-African ancestry, it recommends estimating the dose based on CYP2C9*2 and *3 and VKORC1 variants with the use of one of the available dosing algorithms. CYP4F2*3 genotyping is considered optional, but if detected, a dose increase of 5–10% is recommended. However, for persons of African descent, genotype-guided dosing is recommended only if information on CYP2C9*5, *6, *8 and *11 genotyping is available, and in this case, genotyping of rs12777823 is considered optional. If this additional genotype information is available, the warfarin dose should first be estimated with the use of a warfarin dosing algorithm and then reduced by 15–30% for each CYP2C9*5, *6, *8 or *11 allele, with an additional 15–30% reduction if the rs12777823 variant is detected.

On the other hand, the DPWG guidelines limit recommendations to VKORC1 −1639G>A and CYP2C9*2 and *3 alleles and only provide a decrease in the loading dose. The use of a different starting dose of warfarin is somewhat controversial and plays different roles in different regions of the world, depending on local experience and standards.

The RNPGx group have recommendations for CYP2C9 and VKORC1 gene pairs and warfarin [14]. Their recommendations correspond to international guidelines issued by the CPIC. Thus, they recommend genotyping the variants: VKORC1 −1639G>A, CYP2C9*2 and CYP2C9*3 alleles, on the one hand, before initiating VKA treatment to determine the optimal dose or to guide the prescription of an alternative therapeutic option; and on the other hand, after initiating treatment to explain a bleeding event or resistance to VKAs.

The Canadian Pharmacogenomics Network for Drug Safety (CPNDS) also recommends that VKORC1 (21639G.A), CYP2C9*2, and CYP2C9*3 testing be considered for all patients, including paediatric patients, within the first 2 weeks of therapy or after a bleeding event. Genotyping results should be interpreted using a pharmacogenetic dosing algorithm to estimate the dose needed [26]. The CPNDS clinical recommendation group, in the order of the RNPGx, recommend testing before initiating therapy. If this is not feasible, they recommend that testing be considered if results can be obtained in the first 2 weeks of therapy, as genetic information may still be useful in estimating the maintenance dose. After 2 weeks, the benefits derived from genetic testing diminish.

The recently published ESC position paper states that prospective genotyping prior to warfarin initiation is advisable. Their recommendation is to genotype for all the relevant CYP2C9, VKORC1 and CYP4F2 alleles irrespective of patient’s ethnicity and adjust the initial dosage with a universal algorithm, that still needs to be developed and properly validated [10].

- Acenocoumarol/phenprocoumon and VKORC1

Same as warfarin, other coumadin derivatives, such as acenocoumarol, phenprocoumon or fluindione, exert their anticoagulant action by inhibiting VKORC1. Therefore, mutations in the VKORC1 gene that lead to reduced production of the VKORC1 protein would also require lower VKA doses needed to achieve the desired INR. However, available pharmacogenetic studies are limited compared to warfarin, due to the fact that the use of these derivatives is highly variable across different countries. In Europe for instance, warfarin has been reported to be used predominantly in the UK and Italy, phenprocoumon in Germany, acenocoumarol in Spain, and fluindione in France [54]. Contrarily, in the US, warfarin is the only VKA commercialized.

For this reason, recommendations on other VKAs are only provided by the European consortia. The DPWG provides therapeutic recommendations for acenocoumarol and fenprocoumon [13]. For both drugs, patients with VKORC1 −1639 AA genotype should receive 50% of the standard initial dose and undergo more frequent INR monitoring. RNPGx extends the recommendation to consider genetic factors to establish warfarin dosage to other VKAs but makes no specific statement regarding treatment adjustment.

Spain’s summary of product characteristics for acenocoumarol (in Spain acenocoumarol is the most commonly used VKA) does advise that patients with CYP2C9 variants *2 and *3 have diminished clearance and can consequently need lower acenocoumarol doses, but again no specific dose adjustments are recommended [55].

### 2.2. Antiplatelets

Four antiplatelet agents in the ATC group B01AC (platelet aggregation inhibitors excluding heparin) are reviewed in the guidelines: acetylsalicylic acid, clopidogrel, prasugrel and ticagrelor. Based on available evidence, therapeutic recommendations are only made for clopidogrel and the CYP2C19 gene by three groups: the CPIC [16], DPWG [17] and RNPGx [14] (Table 3).

- Clopidogrel and CYP2C19

CYP2C19 is the main enzyme involved in the conversion of clopidogrel into its active form that acts irreversibly inhibiting the P2Y12 receptor, thus preventing platelet activation and aggregation [56]. CYP2C19 presents significant genetic variation, with approximately 30% of individuals presenting reduced or absent enzyme activity due to genetic polymorphisms. The most frequent no-function alleles are *2 (~15% in Caucasians and Africans, and 29–35% in Asians) and *3 (2–9% in Asians) (others include *4, *5, *6, *7 and *8 but are very rare). There are also alleles associated with increased enzyme function, among which the most common is *17 (3–21% in different ethnicities) [16]. Alleles *2, *3 and *17 are those backed up by a bigger body of scientific evidence, which is why they are the ones considered essential for testing by the US Association for Molecular Pathology [57]. Both the DPWG and RNPgx base their recommendations on the metabolic status inferred by the determination of these three alleles. CPIC considers other possible no-function alleles in case they are present, but recognizes they are rarely determined and even more rarely found.

The Dutch Pharmacogenetics Working Group considers genotyping before starting clopidogrel to guide drug and dose selection in percutaneous coronary intervention or stroke patients to be essential for drug efficacy. The RNPGx considers that a priori genotyping is essential for coronary angioplasty with stenting and potentially useful in other indications.

All three groups agree to recommend the switch to an alternative platelet inhibitor not metabolized by CYP2C19 (e.g., prasugrel, ticagrelor) for both the intermediate (IM) and poor metabolizer (PM) status, due to the risk of treatment ineffectiveness and consequent thrombotic events.

Prasugrel and ticagrelor also act by inhibiting the P2Y12 receptor but have significantly different pharmacokinetic and pharmacodynamic characteristics, with a quicker and more potent antiplatelet effect and less variability in response. However, they are also associated with a higher risk of bleeding when compared with clopidogrel [58]. An important limitation to the clopidogrel switching recommendation is that all three P2Y12 inhibitors are not interchangeable in all clinical situations. For example, the more potent alternatives (prasugrel, ticagrelor) are usually preferred in high-risk patients undergoing Percutaneous Coronary Intervention (PCI), whereas clopidogrel is the only P2Y12 antagonist indicated for established peripheral artery disease and long-term secondary prevention of stroke, and also the only one that can be used in combination with anticoagulant therapy [59,60,61]. For this reason, the CPIC guidelines focus their recommendations on patients with acute coronary syndrome undergoing PCI, for whom more evidence is available. The DPWG considers that alternative agents should be used in CYP2C19 IM and PM for stroke and transient ischemic attack as well as PCI. It should be noted that prasugrel and ticagrelor cannot be used in these circumstances and alternatives to clopidogrel are limited (e.g., dipyridamole). For other indications, measuring the level of platelet inhibition may be considered in PM to warrant minimal drug effectiveness. In the case of intermediate metabolizers, doubling the dose to 150 mg/day and a 600 mg loading dose can also be considered. The 2010 FDA Drug Safety Communication on the use of clopidogrel in PM warns that although the use of a higher dose regimen increases antiplatelet response, the appropriateness of this regimen has not been established in a clinical outcome trial [62]. The RNPgx recommends testing for the main CYP2C19 deficiency alleles before instituting clopidogrel treatment (a test is essential for coronary angioplasty with stenting and based on the current state of knowledge this test is potentially useful in the other indications). For patients carrying at least one deficiency allele, the recommendation is to use an alternative treatment that is not a CYP2C19 substrate [14].

Clopidogrel´s European and American SPCs state that alternative treatments may be considered in patients who are CYP2C19 PM due to the risk of treatment failure [59,63]. However, this is not listed as a specific contraindication and no recommendations are made on how to best determine the patient´s metabolic status.

The latest position statement from the ESC on the role of pharmacogenomics in contemporary cardiovascular therapy insists that CYP2C19 genotyping is only recommended in high-risk cardiovascular situations (e.g., ACS undergoing percutaneous intervention, patients at a high risk of thrombosis or bleeding or patients with recurrent adverse events) and not to systematically tailor the selection of antiaggregation therapy [10]. However, it does recognize that clopidogrel should be avoided in those patients that are known to be intermediate or poor metabolizers due to the risk of ineffectiveness. The 2020 ESC Guidelines for the management of acute coronary syndrome in patients presenting without persistent ST-segment elevation also recognize that CYP2C19 genotyping may be useful to de-escalate P2Y12 receptor inhibitor treatment (e.g., in patients deemed unsuitable for potent platelet inhibition) with a Class IIB, level A evidence [64].

2016. ACC/AHA Guideline focused update on duration of dual antiplatelet therapy in patients with coronary artery disease recommends against routine pharmacogenetic testing for clopidogrel on the base no randomized controlled trials have demonstrated that testing improves patients’ outcomes [65]. However, it must be noted that this guideline was published before relevant evidence from randomized control trials was available and is probably due for actualization soon [66,67,68].

### 2.3. Statins

Three drugs pertaining to the ATC group C10AA HMG CoA reductase inhibitors are reviewed in the guidelines: atorvastatin, simvastatin and fluvastatin. Based on available evidence, therapeutic recommendations are made for simvastatin and the SLCO1B1 gene by three groups: the CPIC [22], DPWG [23] and RNPGx [14]. The DPWG group also has recommendations for Atorvastatin and SLCO1B1 [15] (Table 4).

- Simvastatin and SLCO1B1

Statins are the most widely prescribed cholesterol-lowering drugs for the treatment and prevention of cardiovascular disease. These drugs decrease endogenous cholesterol synthesis by inhibiting the enzyme HMG-CoA reductase and the systemic concentration of low-density lipoprotein cholesterol (LDL-C) [69].

The most commonly prescribed is simvastatin. Despite the success of this drug, the use of simvastatin has been associated with an increased risk of myopathy, which is estimated to occur in 1–5% of patients treated with this drug [70].

The risk of myopathy may be partly explained by a genetic variation in the SLCO1B1 gene. This gene encodes organic anion transporter polypeptide 1B1 (OATP1B1), a transporter that is essential for hepatic uptake and subsequent elimination of statins [71]). A genetic polymorphism in SLCO1B1, c.521T>C (rs4149056), is associated with a significant reduction in transporter activity [72], with significantly lower LDL-C reductions and a higher risk of myopathy, especially in homozygous patients [73]. The minor C allele at rs4149056 is contained within SLCO1B1*5, as well as the *15 and *17 haplotypes and is associated with lower plasma clearance of simvastatin [74].

All groups recommend reducing the dose of simvastatin to no more than 20 mg per day or prescribing another statin for patients carrying at least one C allele of the reduced-function rs4149056. If the homozygous variant (CC) is present, the DPWG directly recommends prescribing an alternative drug [23].

The RNPGx considers rs4149056 genotyping potentially useful before starting statin treatment, especially in patients at high risk of myopathy and also when muscle toxicity appears. However, the ESC considers that routine pharmacogenetic testing before statin initiation is not necessary. Only if patients are known homozygous carriers of allele SLCO1B1*5, the maximum simvastatin dosage (80 mg) must be avoided and an alternative statin used whenever possible.

The simvastatin European summary of product characteristics advises that carriers of the SLC01B1 gene c.521T>C allele have lower OATP1B1 activity. The mean exposure (AUC) of the main active metabolite, simvastatin acid, is 120% in heterozygote carriers (CT) of the C allele and 221% in homozygote (CC) carriers relative to that of patients who have the most common genotype (TT). The C allele has a frequency of 18% in the European population. In patients with SLC01B1 polymorphism there is a risk of increased exposure of simvastatin which may lead to an increased risk of rhabdomyolysis. The FDA drug label only states that simvastatin is a substrate of the transport protein OATP1B1.

- Atorvastatin and SLCO1B1

Regarding atorvastatin, evidence has shown associations between the C allele variant and higher rates of intolerance and muscle-associated adverse effects [75]. Therefore, the DPWG guidelines extend their simvastatin recommendation and advise that atorvastatin should also be avoided in C allele carriers with substantial additional risk factors for myopathy.

### 2.4. Beta-Blockers

Five drugs pertaining to the ATC group C07A BETA BLOCKING AGENTS are reviewed in the DPWG guidelines: atenolol, bisoprolol, carvedilol, metoprolol and sotalol. Among them, recommendations are only published for metoprolol and CYP2D6 (2018) (Table 5).

- Metoprolol and CYP2D6

CYP2D6 is the main enzyme involved in the catabolism of most beta-blockers including carvedilol, metoprolol, nevibolol, propranolol and timolol, and has a relevant role in the metabolism of 20–25% of prescribed drugs [76].

The CYP2D6 gene is highly polymorphic and there is wide interindividual variability in the associated enzyme activity. Specifically, several alleles (*3, *4, *5, etc.) condition null enzyme activity. In contrast, patients with more than two copies of functional alleles (*1/*2) have increased CYP2D6 activity.

The percentage of ultra-rapid metabolizers is variable and depends on ethnic origin.

In addition, CYP2D6 is a gene susceptible to metabolic induction and inhibition by other drugs and compounds which makes phenotype determination even more difficult.

It has been reported that due to increased metoprolol concentrations, CYP2D6 IM and especially PM experience lower heart rates and an increased incidence of bradycardia [77,78,79]. However, this does not seem to relevantly impact drug safety and tolerability in most cases.

The DPWG recommends that when a gradual reduction in heart rate is desired in IM and PM, dose titration should be carried out slowly. If symptomatic bradycardia occurs, it is recommended to use not more than 25–50% of the standard dose.

In the case of UM, targeting the maximum standard dosage is recommended. When the desired effect is not achieved, increasing the dose up to 2.5× the standard dose can be considered with careful motorization of possible adverse events. Use of other beta-blockers not metabolized by CYP2D6 (e.g., atenolol, bisoprolol) or metabolized to a lesser extent (carvedilol) can be considered in these cases depending on indication and other clinical considerations.

At this time, the CPIC considers that evidence supporting the use of genetic evidence to make treatment recommendations for beta-blockers and CYP2D6 is weak (B/C) and has not yet published any guidelines on this issue. No recommendations by the RNPGx are available either.

The ESC working group on cardiovascular pharmacotherapy states that for patients known to be CYP2D6 PM or UM, avoiding metoprolol when starting beta-blocker treatment seems prudent since there are multiple alternatives to choose from. However, no clear statement on when and which patients to genotype is made besides for those patients that suffer metoprolol-related adverse events. They also advise prescribers to be cautious when concomitant treatment with CYP2D6 inhibitors (e.g., bupropion, fluoxetine, paroxetine, and quinidine) is used due to the risk of phenoconversion from the baseline genotype-inferred metabolic status.

European and US metoprolol drug labels acknowledge that CYP2D6 PM show plasmatic metoprolol concentrations significantly higher than normal metabolizers but state that this shows little to no effect in the drug´s safety and tolerability [80].

### 2.5. Antiarrithmics

Four drugs in the ATC group C01B ANTIARRHYTHMICS, CLASS I AND III (disopyramide, flecainide, propafenone and quinidine) are reviewed in the guidelines. Treatment recommendations are available for flecainide and propafenone by the DPWG (2018) (Table 6).

- Flecainide and CYP2D6

Flecainide is indicated for the treatment and prevention of several types of arrhythmias including supraventricular tachicardias and ventricular arrhythmias. It is also used for pharmacological cardioversion and as a provocation test to diagnose Brugada syndrome. It belongs to class IC antiarrhythmic agents, and works by blocking the cardiac fast inward sodium (Na+) current resulting, thus slowing cardiac conduction. Andrikopoulos 25717355.

Flecainide is mainly metabolized via CYP2D6 which is why IM and PM are at risk of drug accumulation and adverse events, due to the drug´s relatively narrow therapeutic range (plasma concentration 200–1000 ng/mL) [18].

Due to this increased risk of adverse events, the DPWG recommends reducing the flecainide dose by half in PM, and by 25% in IM, and monitoring the effect via ECG and plasma levels if possible. The indication for the diagnosis of Brugada syndrome is excluded from this recommendation. In UM caution is advised although data is lacking, and ECG monitoring, plasma levels or the use of alternatives that are not CYP2D6 substrates (e.g., sotalol, disopyramide, quinidine) is recommended.

CPIC has not yet issued a guideline, but the clinical annotation on CYP2D6*1, CYP2D6*4, CYP2D6*5, CYP2D6*10, CYP2D6*21, CYP2D6*36 and flecainide is assigned to level 1A of evidence. It recognizes that patients carrying one or more no function or decreased function alleles may have lower clearance of flecainide but advises that other genetic and clinical factors may also influence flecainide metabolism. The annotation only covers the pharmacokinetic relationship, and due to conflicting evidence on clinical outcomes, no treatment recommendations are made. https://www.pharmgkb.org/clinicalAnnotation/1183621726 (accessed on 6 October 2021).

No relevant pharmacogenetic information is included in flecainide drug labels besides that its metabolism appears to involve the cytochrome P450 isoenzyme CYP2D6, which shows genetic variation.

- Propafenone and CYP2D6

Same as flecainide, the propafenone antiarrhythmic effect is mediated by blockage of the fast sodium current. It is also a weak potassium channel blocker and can exert beta- blocking effects. It works by slowing conduction and prolonging refractoriness of cardiac conduction tissue. Propafenone is indicated for life-threatening ventricular arrhythmias, various supraventricular arrhythmias, and atrial fibrillation and has a therapeutic range between 0.2 and 1.5 µg/mL. (Kaplan’s Essentials of Cardiac Anesthesia).

Propafenone is metabolized by CYP2D6, CYP3A4, and CYP1A2 enzymes. Standard doses of propafenone will lead to higher plasma drug concentrations in poor metabolizers, compared to normal metabolizers. In addition, drugs that inhibit CYP2D6, CYP3A4, and CYP1A2 may also increase propafenone levels, which may lead to cardiac arrhythmia episodes [81].

The DPWG recommends a 70% reduction of the standard dose in PM. For IM and UM, data is lacking in order to recommend specific dose adjustments but the use of an antiarrhythmic drug that is not a CYP2D6 substrate is recommended if possible. The use of ECG and measurement of plasma drug concentrations is recommended in all three cases to monitor adverse events or possible inefficacy [21].

The FDA-approved drug label for propafenone states that administration of the drug to CYP2D6′s slow and extensive metabolizers resulted in significant differences in plasma concentrations, but that no dose adjustments are needed.

ESC guidelines for the diagnosis and management of atrial fibrillation only mention that CYP2D6 inhibitors increase flecainide concentrations but do not mention any effect of genetic variations on treatment effectiveness and safety. No specific mention of propafenone and CYP2D6 is included [82].

Drug labels acknowledge that in CYP2D6 PM propafenone clearance is impaired and plasma half-life is significantly longer. However, no dosing adjustments are recommended in PM patients [83].

### 2.6. Other Drugs Used in Cardiology

One antihypertensive agent in the group C02 ANTIHYPERTENSIVES (clonidine) is reviewed in the guidelines in regard to CYP2D6, but no recommendations are made based on available evidence.

Research for new biomarkers to decrease adverse events of other drugs frequently used in cardiology is ongoing. Many studies have found significant associations between genetic variants and drug toxicity and effectiveness, but in most cases available evidence does not reach the established threshold to warrant publication of specific therapeutic recommendations and pharmacogenetic guidelines.

For instance, CES1 rs2244613 has been associated to higher dabigatran plasma levels and an increased risk of bleeding [84]. Dabigatran is a direct oral anticoagulant widely used for atrial fibrillation instead of vitamin k antagonists. Polymorphisms in CYP2C9 and ABCB1 have been associated to response to angiotensin receptor blocker losartan, since these proteins participate in the drug´s metabolism and transport [85,86]. Also, variant rs1799752 in the gene coding for the Angiotensin Converting Enzyme (ACE) has been linked to the effectiveness of some ACE inhibitors, such as captopril [87] and enalapril, [88] and to spironolactone; patients with del/del genotype and chronic heart failure may have less improvement in left ventricular ejection fraction, end-systolic and end-diastolic volume [89]. Variants in the ACE gene have also been proposed as potential predictors of susceptibility to COVID-19 [90]. A significant association between alpha-adducin gene1 (ADD1) Gly460Trp polymorphism and blood pressure change with hydrochlorothiazide has also been reported [91]. In regard to antiarrhythmics, a polygenic risk score has been proposed to predict the risk of QT prolongation or torsade the pointe induced by quinidine or dofetilide [92].

All these drug-gene pairs are currently assigned a level of evidence that ranges between 2A and 3 according to the PharmGKB scoring system.

## 3. Differences in Therapeutic Recommendations

Genetic information on drug labels is often unspecific or vague and does not usually include recommendations on how to adjust treatment accordingly. Different national and international groups provide evidence-based guidelines to help drug selection and dosage adjustment according to pharmacogenetic results in order to avoid unnecessary adverse events and optimize response.

The US Clinical Pharmacogenetics Implementation Consortium (CPIC), the Dutch Pharmacogenetics Working Group (DPWG), the French Network of Pharmacogenetics (Réseau national de pharmacogénétique (RNPGx) and The Canadian Pharmacogenomics Network for Drug Safety (CPNDS) are probably the most well-known and influential pharmacogenetic consortia in North America and Europe.

Among these groups, the Dutch is the one that has published more recommendations on cardiovascular drug-gene pairs. In most cases, they are also the more recent ones, having been updated in 2018. The CPNDS is the consortia with fewer recommendations in the cardiovascular area, having only issued recommendations for warfarin and CYP2C9.

With the exception of the RNpGx, the guidelines do not often address when and for which patients pharmacogenetic testing must be ordered, leaving that to doctor´s criteria.

Differences in the methodology used by these groups, as well as discrepant allele terminology, classification and phenotype assignment make it even more difficult to compare and select specific recommendations to be used in clinical practice. Differences in clinical practices between countries are also conditioning, as is the case of the use of different VKAs in different countries or unequal access to direct measurement of plasma levels or drug´s effect (e.g., INR monitoring).

Whenever discrepant recommendations are available, considering the country of origin’s clinical practices in relation to yours is advisable. Also, considering which one is more recent can be helpful since new evidence may have arisen and is being incorporated.

The recently published ESC guideline on the role of pharmacogenomics in cardiology marks an important milestone towards clinical implementation. Although cautious in most of its recommendations, the impact the society guidelines have in setting practice standards will undoubtedly help convince many reluctant clinicians. To the best of our knowledge, no specific guidelines on pharmacogenomics have been yet issued by the AHA.

## 4. Conclusions

Cardiovascular drugs are amongst the most widely prescribed medicines in the world, which is why treatment optimization is fundamental to reduce associated morbidity and mortality. Tailoring treatment according to a patient’s genotype has proven effective for a number of drug-gene pairs in order to reduce adverse events and increase effectiveness. However, significant differences exist between recommendations given by Pharmacogenetic Consortia and other relevant Societies. In order to effectively apply this into clinical practice, a wider consensus must be reached and official recommendations on how to adjust treatment based on a patient´s genetic profile must be issued.

## Figures and Tables

**Table 1:** Cardiovascular drug-gene pairs reviewed in main pharmacogenetic guidelines.

Reviewed with Recommendation	Reviewed with No Recommendation
Acenocoumarol-*VKORC1* (DPWG [13], RNPGx [14]) Atorvastatin-*SLCOB1* (DPWG [15]) Clopidogrel-*CYP2C19* (CPIC [16], DPWG [17], RNPGx [14]) Flecainide-*CYP2D6* (DPWG [18]) Metoprolol-*CYP2D6* (DPWG [19]) Phenprocoumon-*VKORC1* (DPWG [20]) Propafenone-*CYP2D6* (DPWG [21]) Simvastatin-*SLCOB1* (CPIC [22], DPWG [23], RNPGx [14]) Warfarin-*CYP2C9* (CPIC [24], DPWG [25], CPNDS [26], RNPGx [14]) Warfarin-*VKORC1* (CPIC [24], DPWG [27], RNPGx [14]) Warfarin-*CYP4F2* (CPIC [24])	Acenocoumarol-*CYP2C9* (DPWG [28]) Amiodarone-*CYP2D6* (DPWG [29]) Aspirin-*CYP2C9* (CPIC [30]) Atenolol-*CYP2D6* (DPWG [31]) Bisoprolol-*CYP2D6* (DPWG [32]) Carvedilol-*CYP2D6* (DPWG [33]) Clonidine-*CYP2D6* (DPWG [34]) Disopyramide-CYP2D6 (DPWG [35]) Fluvastatin-*SLCOB1* (DPWG [36]) Phenprocoumon-*CYP2C9* (DPWG [37]) Prasugrel-*CYP2C19* (DPWG [38]) Quinidine-*CYP2D6* (DPWG [39]) Sotalol-*CYP2D6* (DPWG [40]) Ticagrelor-*CYP2C19* (DPWG [41])

CPIC, Clinical Pharmacogenetics Implementation Consortium; DPWG, Dutch Pharmacogenetics Working Group; RNPGx, French Network of Pharmacogenetics (Réseau national de pharmacogénétique); CPNDS, Canadian Pharmacogenomics Network for Drug Safety.

**Table 2:** Drug-gene pairs and dose recommendations by different clinical guidelines. Anticoagulants.

Drug	Gene	Guideline	Genotype/Phenotype	Therapeutic Recommendation	Level of Evidence
Warfarin	*CYP2C9* Combined with *VKORC1* and *CYP4F2*	CPIC 2017	*1/*2	Use validated pharmacogenetics algorithms to calculate initial dose	Strong (Non-African) Moderate (African)
			*1/*3		
			*2/*2		
			*2/*3		
			*3/*3		
Warfarin	*CYP2C9*	DPWG 2018	*1/*2 (IM)	Use initial standard dose	4A
			*1/*3 (IM)	Use 65% of the standard initial dose. Specific dose can be calculated using an algorithm	4D
			*2/*2 (PM)	Use 65% of the standard initial dose. Specific dose can be calculated using an algorithm	4A
			*2/*3 (PM)	Use 45% of the standard initial dose. Specific dose can be calculated using an algorithm	4A
			*3/*3 (PM)	Use 20% of the standard initial dose. Specific dose can be calculated using an algorithm	4C
Warfarin	*CYP2C9* Combined with *VKORC1*	RNPGx 2017	*1/*1	Suggested initial dose between 5 and 7 mg or 3 and 4 mg depending on *VKORC1* genotype	A priori genotyping: *advisable* A posteriori: *advisable*
			*1/*2 (IM)	Suggested initial dose between 5 and 7 mg or 3 and 4 mg depending on *VKORC1* genotype	
			*1/*3 (IM)	Suggested initial dose between 3 and 4 mg or 0.5 and 2 mg depending on *VKORC1* genotype	
			*2/*2 (PM)	Suggested initial dose between 3 and 4 mg or 0.5 and 2 mg depending on *VKORC1* genotype	
			*2/*3 (PM)	Suggested initial dose between 3 and 4 mg or 0.5 and 2 mg depending on *VKORC1* genotype	
			*3/*3 (PM)	Suggested initial dose between 0.5 and 2 mg	
Warfarin	*CYP2C9* Combined with *VKORC1*	CPNDS 2015	*2	Use pharmacogenetic dosing algorithm to estimate the required dose	Strong A
			*3		
Warfarin	*VKORC1* Combined with *CYP2C9* and *CYP4F2*	CPIC 2017	−1639 AG	Use validated pharmacogenetics algorithms to calculate initial dose	Strong (Non-African) Moderate (African)
			−1639 GG		
Warfarin	*VKORC1*	DPWG 2018	−1639 AG	Use initial standard dose	4A
			−1639 GG	Use 60% of the standard initial dose. Specific dose can be calculated using an algorithm	4A
Warfarin	*VKORC1* Combined with *CYP2C9*	RNPGx 2017	−1639 GG	Suggested initial dose between 5 and 7, 3 and4 or 0.5 and 2 mg depending on *VKORC1* genotype	1A
			−1639 AG	Suggested initial dose between 5 and7, 3and 4 or 0.5 and 2 mg depending on *VKORC1* genotype	
			−1639 AA	Suggested initial dose between 5–7, 3–4 or 0.5–2 mg depending on *VKORC1* genotype	
Warfarin	*VKORC1* Combined with *CYP2C9*	CPNDS 2015	−1639 AG	Use pharmacogenetic dosing algorithm to estimate the required dose	Strong A
			−1639 GG		
Warfarin	*CYP4F2* Combined with *CYP2C9* and *VKORC1*	CPIC 2017	rs2108622 T	Increase initial dose calculated with algorithm by 5–10%	Optional
Acenocoumarol	*VKORC1*	DPWG 2018	−1639 AG	Use initial standard dose	4C
			−1639 AA	Use 50% of the standard initial dose. Recommend more frequent INR monitoring	4F
Phenprocoumon	*VKORC1*	DPWG 2018	−1639 AG	Use initial standard dose	4D
			−1639 AA	Use 50% of the standard initial dose. Recommend more frequent INR monitoring	4D

CPIC, Clinical Pharmacogenetics Implementation Consortium; DPWG, Dutch Pharmacogenetics Working Group; RNPGx, French Network of Pharmacogenetics (Réseau national de pharmacogénétique); CPNDS, Canadian Pharmacogenomics Network for Drug Safety; IM, Intermediate metabolizer; PM, Poor metabolizer; INR, international normalized ratio.

**Table 3:** Drug-gene pairs and dose recommendations by different clinical guidelines. Antiplatelets.

Drug	Gene	Guideline	Genotype/Phenotype	Therapeutic Recommendation	Level of Evidence
Clopidogrel	*CYP2C19*	CPIC 2013	UM (*1/*17 or *17/*17)	Label recommended dosage and administration	Strong
			EM (*1/*1)	Label recommended dosage and administration	Strong
			IM (*1/*2; *1/*3 or *2/*17)	Alternative antiplatelet therapy (if no contraindication)	Moderate
			PM (*2/*2; *2/*3 or *3/*3)	Alternative antiplatelet therapy (if no contraindication)	Strong
Clopidogrel	*CYP2C19*	DPWG 2018	UM (*17/*17)	Label recommended dosage and administration	4A
			IM (*1/*2; *1/*3; *2/*17 or *3/*17)	Percutaneous coronary intervention, stroke or TIA: choose an alternative or double the dose to 150 mg/day (600 mg loading dose). Other indications: no action required	4F
			PM (*2/*2; *2/*3 or *3/*3)	Percutaneous coronary intervention, stroke or TIA: choose an alternative or double the dose to 150 mg/day (600 mg loading dose). Other indications: determine the level of inhibition of platelet aggregation by clopidogrel. Consider an alternative in poor responders	4F
Clopidogrel	*CYP2C19*	RNPGx 2017	UM (*1/17 or *17/*17)	Label recommended dosage and administration	A priori genotyping: Coronary angioplasty: *essential* Other: *potentially useful* A posteriori: *advisable*
			EM (*1/*1)	Label recommended dosage and administration	
			IM (*1/*2 or *1/*3)	Alternative antiplatelet therapy	
			PM (*2/*2; *3/*3)	Alternative antiplatelet therapy	

CPIC, Clinical Pharmacogenetics Implementation Consortium; DPWG, Dutch Pharmacogenetics Working Group; RNPGx, French Network of Pharmacogenetics (Réseau national de pharmacogénétique); UM, Ultrarapid metabolizer; EM, Extensive metabolizer; IM, Intermediate metabolizer; PM, Poor metabolizer; TIA, Transient ischaemic attack.

**Table 4:** Drug-gene pairs and dose recommendations by different clinical guidelines. Statins.

Drug	Gene	Guideline	Genotype/Phenotype	Therapeutic Recommendation	Level of Evidence
Simvastatin	*SLCO1B1*	CPIC 2014	IM (CT)	Lower dose or consider an alternative statin (consider routine CK surveillance)	Strong
			PM (CC)	Lower dose or consider an alternative statin (consider routine CK surveillance)	Strong
Simvastatin	*SLCO1B1*	DPWG 2020	521 CT	1. Choose an alternative 2. If not possible: (a) Avoid simvastatin doses exceeding 40mg/day. (b) Advise the patient to contact their doctor in the event of muscle symptoms.	4D
			521 CC	Choose an alternative drug	4D
Simvastatin	*SLCO1B1*	RNPGx 2017	521 TT	Avoid maximum dose (80 mg) during the first year of treatment	A priori genotyping: *no indication* A posteriori: *potentially useful*
			521 CT	Reduce the dose to max. 20 mg per day. Close CPK monitoring and avoid OATP1B1 inhibitors	
			521 CC	Reduce the dose to max. 20 mg per day. Close CPK monitoring and avoid OATP1B1 inhibitors	
Atorvastatin	*SLCO1B1*	DPWG 2020	521 CT	Additional risk factors for myopathy: 1. Choose an alternative 2. If not possible: Advise the patient to contact their doctor in the event of muscle symptoms. No additional risk factors for myopathy: Advise the patient to contact their doctor in the event of muscle symptoms.	4C
			521 CC	Additional risk factors for myopathy: 1. Choose an alternative 2. If not possible: Advise the patient to contact their doctor in the event of muscle symptoms. No additional risk factors for myopathy: Advise the patient to contact their doctor in the event of muscle symptoms.	4C

CPIC, Clinical Pharmacogenetics Implementation Consortium; DPWG, Dutch Pharmacogenetics Working Group; RNPGx, French Network of Pharmacogenetics (Réseau national de pharmacogénétique); IM, Intermediate metabolizer; PM, Poor metabolizer; CPK, Creatine Phosphokinase.

**Table 5:** Drug-gene pairs and dose recommendations by different clinical guidelines. Beta-blockers.

Drug	Gene	Guideline	Genotype/Phenotype	Therapeutic Recommendation	Level of Evidence
Metoprolol	*CYP2D6*	DPWG 2018	UM	1. Use the maximum dose for the relevant indication as a target dose. 2. If effectiveness is still insufficient: increase the dose based on effectiveness and side effects to 2.5 times the standard dose or select an alternative.	4D
			IM	Gradual reduction of heart rate or in the event of symptomatic bradycardia: 1. increase the dose in smaller steps and/or prescribe no more than 50% of the standard dose. Other cases: no action required.	4A
			PM	Gradual reduction of heart rate or in the event of symptomatic bradycardia: 1. increase the dose in smaller steps and/or prescribe no more than 25% of the standard dose. Other cases: no action required.	4C

DPWG, Dutch Pharmacogenetics Working Group; UM, Ultrarapid metabolizer; IM, Intermediate metabolizer; PM, Poor metabolizer.

**Table 6:** Drug-gene pairs and dose recommendations by different clinical guidelines. Antiarrythmics.

Drug	Gene	Guideline	Genotype/Phenotype	Therapeutic Recommendation	Level of Evidence
Flecainide	*CYP2D6*	DPWG 2018	UM	Monitor the plasma concentration as a precaution and record an ECG or select an alternative	NA
			IM	Indications other than diagnosis of Brugada syndrome: reduce the dose to 75% of the standard dose and record an ECG and monitor the plasma concentration. Provocation test for diagnosis of Brugada syndrome: No action required.	3A
			PM	Reduce the dose to 50% of the standard dose and record an ECG and monitor the plasma concentration.	4F
Propafenone	*CYP2D6*	DPWG 2018	UM	Monitor the plasma concentration as a precaution and record an ECG or select an alternative (possible reduced efficacy)	3D
			IM	Monitor the plasma concentration as a precaution and record an ECG or select an alternative (be alert to side effects)	3A
			PM	Reduce the dose to 30% of the standard dose, perform an ECG and monitor plasma concentrations.	4C

DPWG, Dutch Pharmacogenetics Working Group; UM, Ultrarapid metabolizer; IM, Intermediate metabolizer; PM, Poor metabolizer; ECG, electrocardiogram; NA, not applicable.
